# Environmentally Tough and Stretchable MXene Organohydrogel with Exceptionally Enhanced Electromagnetic Interference Shielding Performances

**DOI:** 10.1007/s40820-022-00819-3

**Published:** 2022-03-21

**Authors:** Yuanhang Yu, Peng Yi, Wenbin Xu, Xin Sun, Gao Deng, Xiaofang Liu, Jianglan Shui, Ronghai Yu

**Affiliations:** 1 School of Materials Science and Engineering, Beihang University, Beijing, 100191 People’s Republic of China; 2 Science and Technology on Optical Radiation Laboratory, Beijing Institute of Environmental Features, Beijing, 100854 People’s Republic of China; 3 Science and Technology on Electromagnetic Scattering Laboratory, Beijing Institute of Environmental Features, Beijing, 100854 People’s Republic of China

**Keywords:** Electromagnetic interference shielding, MXene organohydrogel, Stretchable conductive film, Anti-drying ability, Low-temperature tolerance

## Abstract

Conductive hydrogels have potential applications in shielding electromagnetic (EM) radiation interference in deformable and wearable electronic devices, but usually suffer from poor environmental stability and stretching-induced shielding performance degradation. Although organohydrogels can improve the environmental stability of materials, their development is at the expense of reducing electrical conductivity and thus weakening EM interference shielding ability. Here, a MXene organohydrogel is prepared which is composed of MXene network for electron conduction, binary solvent channels for ion conduction, and abundant solvent-polymer-MXene interfaces for EM wave scattering. This organohydrogel possesses excellent anti-drying ability, low-temperature tolerance, stretchability, shape adaptability, adhesion and rapid self-healing ability. Two effective strategies have been proposed to solve the problems of current organohydrogel shielding materials. By reasonably controlling the MXene content and the glycerol-water ratio in the gel, MXene organohydrogel can exhibit exceptionally enhanced EM interference shielding performances compared to MXene hydrogel due to the increased physical cross-linking density of the gel. Moreover, MXene organohydrogel shows attractive stretching-enhanced interference effectiveness, caused by the connection and parallel arrangement of MXene nanosheets. This well-designed MXene organohydrogel has potential applications in shielding EM interference in deformable and wearable electronic devices.

**Supplementary Information:**

The online version contains supplementary material available at 10.1007/s40820-022-00819-3.

## Highlights

- Stretchable MXene organohydrogel contains MXene network for electron conduction and water/glycerin binary solvent for ion transmission was prepared.
- The MXene organohydrogel exhibits exceptionally enhanced EMI shielding performance compared to hydrogel, as well as low-temperature tolerance, anti-drying ability.

**Supplementary Information:**

The online version contains supplementary material available at 10.1007/s40820-022-00819-3.

## Graphical abstract

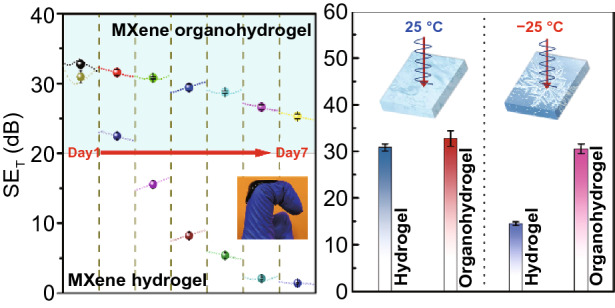

## Introduction

Nowadays, deformable and wearable electronic devices integrated with the Internet of things (IoT) are constantly refreshing the development of society and technology [1–4]. However, the electromagnetic radiation generated in the process of transmitting and receiving electromagnetic waves may interfere with the normal operation of the devices, cause information leakage and even threaten human health [5–8]. Highly efficient electromagnetic interference (EMI) shielding materials toward gigahertz frequency are essential to suppress or control the undesirable EM radiation [9, 10]. So far, various types of shielding materials have been developed, such as metal film/mesh [11–13], carbonaceous foam [14–16], MXene aerogel/film [17–21]. Nevertheless, the current EMI shielding materials prepared based on highly conductive materials still face the challenges of simultaneously possessing flexibility, stretchability, biolocompatibility and high responsibility to external stimuli, which limit their applications in deformable and wearable electronic devices [13, 22]. In addition, once fabricated, these shielding materials cannot be re-edited, recycled, and recovered from damage, causing massive waste worldwide.

Conductive hydrogels with excellent conductivity, flexibility, stretchability and remarkable biological characteristics are receiving tremendous attention for their substantial applications in electronic skins, soft robotics, sensors, supercapacitors and so on [23, 24]. These characteristics endow hydrogels with potentials for providing EM radiation protection for deformable and wearable electronics. Compared to the other EMI shielding materials, hydrogels have unique advantages, that is, the water confined in the hydrogel network is capable of generating strong polarization loss to attenuate gigahertz EM waves [25]; at the same time, hydrogels could transmit current through ions in the water phase. In addition, the large deformability and self-healing ability of hydrogels are very important for the recycling of shielding materials and preventing EM protection failures caused by mechanical damage. Despite these attractive advantages, efficient gigahertz EMI shielding hydrogels have barely been reported because of the poor environmental stability. With pure water as dispersion medium, the hydrogel shielding materials will inevitably suffer from water evaporation and sub-zero freezing, which limit their practical applications and shorten their service life. To solve these problems, organohydrogels have been developed by introducing organic agents such as ethylene glycol (EG), glycerol (Gly), or sorbitol into hydrogels [26, 27]. Unfortunately, the conductivity and dielectric loss of the organohydrogels, which are essential factors determining EMI shielding ability, are reduced with the decreasing of water content [28, 29]. Therefore, it is generally believed that the EMI shielding ability of organohydrogels is weaker than that of hydrogels. Nowadays, how to obtain excellent environmental adaptability while maintaining the high EMI shielding performance is an urgent challenge to be solved for organohydrogel.

Moreover, the conductivity of organohydrogel generally decreases during stretching. For example, ionically conductive organohydrogel (LiCl-loaded PAA-based organohydrogel) showed a linear increase of relative resistance variation (Δ*R*/*R*_0_) with tensile strain, which was attributed to the elongated and narrowed transport path of ions under stretching deformation [30]. Similarly, the Δ*R*/*R*_0_ value of CNTs-filled organohydrogel monotonously increased with the increase of tensile strain due to the damage of the continuous CNT network [23]. The decrease of conductivity of organohydrogel during stretching will weaken the reflection and absorption of materials to EM waves. If integrated into deformable and wearable electronics, the shielding performance of the traditional organohydrogel will decrease when the device is bent and stretched, which is another concern for the application of organohydrogel shielding material.

In previous studies, the design of highly efficient EMI shielding materials has focused on improving the electrical conductivity or polarization relaxation of the materials to enhance the reflection and absorption of EM waves [31, 32]. From this point of view, it is difficult to solve the problem of performance degradation of organohydrogel relative to hydrogel. Therefore, it is necessary to find new strategy to improve the shielding performance of gel. Although it has been recognized that the porous structure of materials can promote the multiple scattering and reflection of EM waves [17, 18, 33], the effect of these physical effects on improving the shielding performance is still underestimated, because the contribution of the porous structure is usually mixed with the contribution of the conductivity and is difficult to distinguish. An improved understanding of the relationship between the porous structure and performance of shielding materials is critical to advancing the design and development of shielding materials, especially in solving the problem of organohydrogel.

Here, we have prepared a MXene organohydrogel containing glycerol (Gly) and water binary solvent, which shows excellent low-temperature tolerance, anti-drying ability, high stretchability, shape adaptability, adhesion and rapid self-healing ability. It is surprising to find that by adjusting the water-Gly ratio in gel, MXene organohydrogel can achieve an exceptional enhancement of EMI shielding performance compared with MXene hydrogel due to the significant increase in the physical cross-linking density of gel. Moreover, the MXene organohydrogel exhibits excellent EMI shielding ability within 100% tensile strain, and shows unusual strain-enhanced shielding effectiveness, which is associated with the parallel orientation and connection of MXene nanosheets and the resultant enhancement of internal electric field and conductivity. In terms of mechanism, we emphasized the key role of increasing cross-linking density of gel in improving the shielding performance, which paves the way for the development of high-performance EMI shielding organohydrogel.

## Experimental Section

### Materials

Ti_3_AlC_2_ powder was purchased from Eleven Technology Company (400 mesh, molecular weight = 194.8, density = 4.2 g cm^−1^). Polyvinyl alcohol (PVA, Mw ~ 195,000), glycerol (Gly, AR, purity ≥ 99%), sodium tetraborate docahydrate (borax, GR, purity ≥ 99.9%), lithium fluoride (LiF, AR, purity ≥ 99%), acrylamide (AAm, AR, purity ≥ 99.0%), N, N′-methylene-bis-acrylamide (MBAA, AR), ammonium peroxodisulfate (APS, AR, purity ≥ 98%) and concentrated hydrochloric acid (AR, 37%) were purchased from Macklin. All reagents were used without further purification.

### Synthesis of MXene nanosheets

LiF (0.5 g) was mixed with HCl (9 M, 10 mL) under stirring, followed by adding Ti_3_AlC_2_ powder (0.5 g) into the solution. The mixture was stirred at 35 °C for 24 h for etching reaction. After cleaning with deionized water, the etched products were sonicated under argon flow. Finally, the dispersion was centrifuged and the supernatant was collected.

### Synthesis of MXene Hydrogel

Polyvinyl alcohol (PVA, 0.2 g) and acrylamide (AAm, 0.7 g) were dispersed in deionized water (5 mL) and the solution was stirred at 90 °C for 2 h. Subsequently, a certain amount of MXene (0.9, 1.7, 3.3, 10.0, and 20.0 mg), N,N′-methylene-bis-acrylamide solution (MBAA, 1 wt%, 60 μL), ammonium peroxodisulfate solution (APS, 10 wt%, 90 μL) and borax solution (4 wt%, 600 μL) were added into the above solution and stirred until a gel formed. The pH value was controlled to be 9.5. In-situ polymerization was carried out at 60 °C to obtain MXene hydrogel. The weight percent of MXene to the sum of PVA and AAM, i.e., *W*_MXene_/(*W*_PVA_ + *W*_AAM_), is 0.1, 0.2, 0.37, 1.1, and 2.2 wt%.

### Conversion of MXene Hydrogel to MXene Organohydrogel

MXene organohydrogel was prepared by solvent displacement method. The above MXene hydrogels were immersed in a mixed solution of glycerol (Gly) and water at room temperature. A series of MXene organohydrogels were prepared by changing the water-Gly ratio (4:1, 1:1, 0:1) and immersion time.

### Characterizations

X-ray diffraction (XRD) analysis was performed on an x-ray diffractor (Bruker D8 Advance). The microstructure of the samples was characterized using scanning electron microscopy (SEM, JEOL-JSM7500) and transmission electron microscopy (TEM, JEOL-JEM2100). For SEM observation, MXene hydrogel and MXene organohydrogel were freeze-dried and quenched using liquid nitrogen to produce fresh cross-sections. The conductivity of the samples (diameter of 25–30 mm, thickness of 3–4 mm) was measured using four-point probe method. Tensile tests were conducted using a universal testing machine (Instron 5565, 5KN). The size of the samples for tensile tests was 20 × 10 × 3 mm^3^.

### Measurement of EMI Shielding Performance

EMI shielding performances of the samples were tested by a vector network analyzer (N5234B PNA-L, KEYSIGHT) in the frequency range of 8.2–12.4 GHz. MXene hydrogel and MXene organohydrogel were cut into rectangles of 22.86 × 10.16 × 1.0 mm^3^. EMI shielding effectiveness is defined as the logarithmic ratio of incident power to transmission power. The total SE (SE_T_) is the summary of reflection effectiveness (SE_R_), absorption effectiveness (SE_A_) and multiple reflection effectiveness (SE_M_). SE_M_ can be ignored when SE is larger than 15 dB. Scattering parameters (*S*_11_, *S*_21_, *S*_21_ and *S*_22_) were measured and used to calculate SE_T_, SE_A_ and SE_R_ according to the following equations [34, 35]:

1 $${\text{SE}}_{{\text{T}}} = 10{\text{log}}\left( {\frac{1}{{\left| {S_{21} } \right|^{2} }}} \right)$$

2 $${\text{SE}}_{{\text{R}}} = 10{\text{log}}\left( {\frac{1}{{1 - |S_{11} |^{2} }}} \right)$$

3 $${\text{SE}}_{{\text{A}}} = 10{\text{log}}\left( {\frac{{1 - |S_{11} |^{2} }}{{\left| {S_{21} } \right|^{2} }}} \right)$$

The absorption (*A*), reflection (*R*), and transmission (*T*) coefficients were calculated as:

4 $$T = |S_{12} |^{2} = |S_{21} |^{2}$$

5 $$R = |S_{11} |^{2} = |S_{22} |^{2}$$

6 $$A + R + T = 1$$

## Results and Discussion

### Synthesis and Characterization of MXene Organohydrogel

**Fig. 1:**
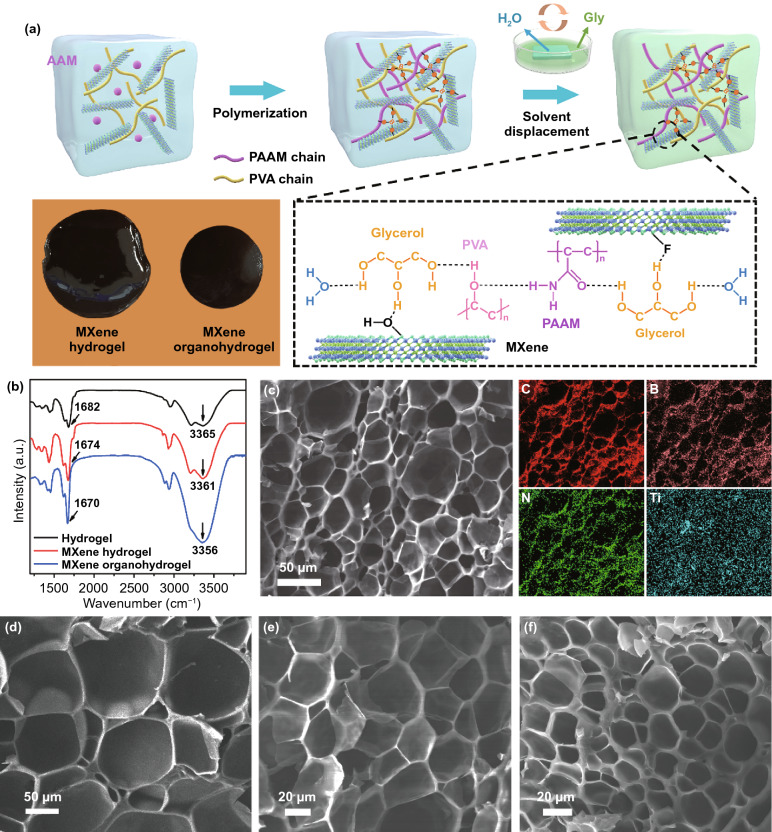
**a** Scheme of the synthesis of MXene hydrogel and organohydrogel, and photos of MXene hydrogel and organohydrogel. **b** FT-IR spectra of pure hydrogel, MXene hydrogel and MXene organohydrogel. **c** Elemental mapping images of MXene organohydrogel (MXene content of 0.4 wt%, Gly displacement time of 30 min). Cross-sectional SEM images of freeze-dried MXene organohydrogel (MXene content of 0.4 wt%) with different Gly displacement time (Gly: water = 1:1): **d** 0 min, **e** 30 min, **f** 60 min

Figure 1a illustrates the synthesis of MXene hydrogel and its conversion to MXene organohydrogel. First, titanium carbide MXene (Ti_3_C_2_T_x_) nanosheets were prepared by selective etching and delamination of MAX (Ti_3_AlC_2_) powder. XRD pattern of MXene nanosheets shows a sharp peak at 5.82°, corresponding to an enlarged interlayer distance of 1.72 nm as compared with that of Ti_3_AlC_2_ (0.96 nm) (Fig. S1) [36]. No diffraction peaks from Ti_3_AlC_2_ were detected, suggesting the successful exfoliation of MXene nanosheets. The ultrathin MXene nanosheet has a few-layer structure with a lateral size of several micrometers (Fig. S2). Subsequently, the MXene nanosheets were mixed with acrylamide (AAm) monomers and polyvinyl alcohol (PVA) in deionized water under stirring, followed by the addition of a chemical crosslinker (N, N′-methylene-bis-acrylamide, MBAA), an initiator ammonium peroxodisulfate solution (APS), and sodium tetraborate decahydrate (borax) to form dynamic cross-linking between the hydroxyl group of PVA and tetrahydroxyl borate ions [37]. Due to the abundant hydrophilic functional groups on the surface, MXene nanosheets were well dispersed in the solution and entangled with polymer chains. Then, the above “clay” was kept at 60 °C for in-situ polymerization to form MXene hydrogel (Fig. 1a). Finally, the MXene hydrogel was immersed in a mixed solution containing water and glycerol (Gly) (volume ratio = 1:1) for solvent displacement. Due to the concentration gradient, molecular exchange occurred rapidly until a dynamic equilibrium was reached, and the volume of the obtained gel shrank (Fig. 1a). Considering the rapid displacement speed, we used a mixture of Gly and water instead of pure Gly as the displacement solvent, which facilitates controlling the amount of Gly in the MXene organohydrogel. During this process, part of “free water” molecules in the hydrogel were displaced by Gly molecules, forming abundant hydrogen bonds with PAAM, PVA and MXene, while the “intermediate water” and “nonrotational bound water” were difficult to be displaced due to the formation of hydrogen bonds with the polymer network [38]. The content of Gly molecules in the organohydrogel can be well tuned by controlling the volume ratio of water to Gly (4:1, 1:1, 0:1) as well as the displacement time. In addition, we prepared several MXene hydrogels with varying weight percent of MXene to the sum of PVA and AAM (0.1, 0.2, 0.4, 1.1, and 2.2 wt%).

MXene organohydrogel was constructed by chemical cross-linking generated between MBAA and PAAM, as well as multiple physical cross-linkings through borax, MXene nanosheets and Gly. Borax was hydrolyzed to generate negatively charged borate ions, which could increase the cross-linking density by producing ionic coordinations with the hydroxyl groups in PVA [39]. MXene nanosheets acted as physical cross-linking points to strengthen network due to the unique two-dimensional lamellar structure with surficial bonded radicals. In addition, Gly with multiple hydroxyl groups formed abundant hydrogen bondings with water and polymer chains, further increasing the cross-linking density. According to the EMI shielding performance discussed below, the optimal MXene content is 0.4 wt% and the optimal displacement time is 30 min (water: Gly = 1:1). Therefore, the optimal MXene hydrogel and corresponding organohydrogel were used as representative samples for the following microstructure characterizations.

Fourier transform-infrared (FT-IR) spectra were characterized to clarify the intermolecular interaction in the MXene hydrogel and organohydrogel (Fig. 1b). In the spectrum of pure hydrogel, the peaks at 3365 and 3214 cm^−1^ represent the N–H stretching vibrations in PAAM. At the same time, the characteristic peak at ~ 3410 cm^−1^ from the O–H stretching vibration of PVA overlaps in this region. In addition, the strong band at 1682 cm^−1^ is attributed to the vibration absorption of C=O bonds in PAAM [40, 41]. The incorporation of MXene nanosheets shifted these peaks to 3361 and 1674 cm^−1^ due to the enhanced energy dissipation during stretching in MXene hydrogel, which suggests the formation of hydrogen bonding between polymer chains and nanosheets. Gly displacement further shifted these peaks to lower wavenumbers of 3356 and 1670 cm^−1^ in MXene organohydrogel, demonstrating that the hydrogen bond interaction between polymer chains and Gly became stronger [23]. The microstructure and elemental distribution of freeze-dried MXene hydrogels and organohydrogels were characterized by SEM. All the samples exhibit 3D interconnected porous microstructure (Fig. 1c–f and S3-S5). Energy dispersive spectrometer (EDS) mapping images reveal the uniform distribution of polymer, MXene and borate ions in MXene hydrogel and organohydrogel (Figs. S3 and 1c). The increase of MXene content decreases the pore size and the thickness of cell wall of MXene hydrogels (Fig. S4). Pure hydrogel has smooth cell walls, while with the increase of MXene content, the cell walls become rougher and rougher, and obvious aggregation of MXene nanosheets could be observed for the MXene hydrogel with MXene content of 2.2 wt% (Fig. S5). Compared to MXene hydrogel, MXene organohydrogel has a more compact pore structure, and the pores size decreases with the increase of Gly displacement time (Fig. 1d–f). These phenomena reveal the increase of cross-linking density of polymer network by MXene addition and Gly displacement [29, 42–44].

### Multifunctional Properties of MXene Organohydrogel

**Fig. 2:**
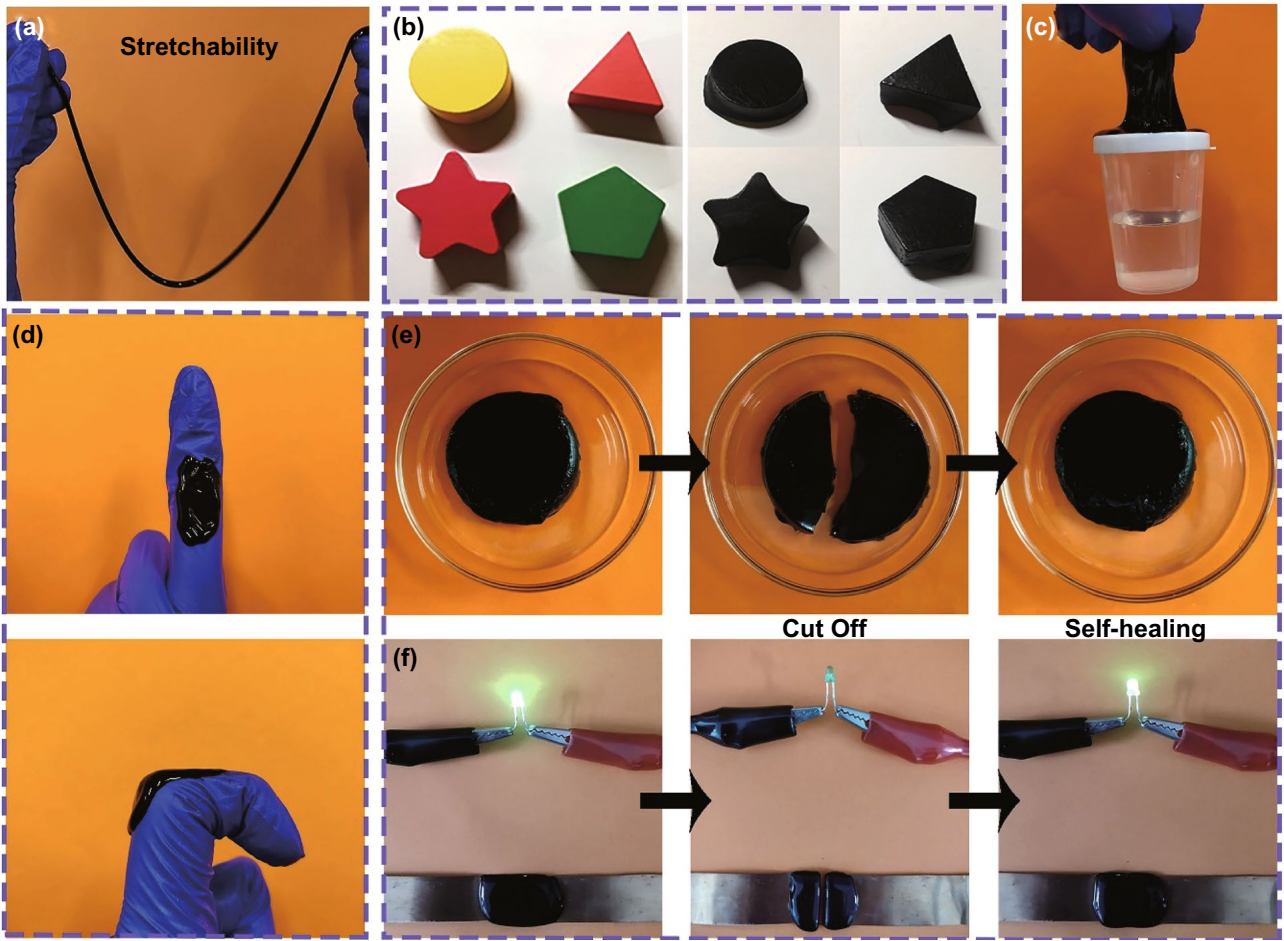
Photographs showing the **a** stretchability, **b** shape adaptability, **c, d** adhesiveness and **e, f** self-healing function of MXene organohydrogel

Chemical cross-linking and reversible physical cross-linking endow MXene organohydrogel with super mechanical properties. Meanwhile, the MXene nanosheets, attached to the cell walls of the gel, can effectively enhance the electrical conductivity of the polymer network. Therefore, the MXene organohydrogel shows excellent stretchability, deformability, adhesion and electrical responsibility. As shown in Fig. 2a, MXene organohydrogel can be repeatedly stretched into a thread with a tensile strain. Figure S6 shows the stress–strain curves of pure hydrogel, MXene hydrogel and MXene organohydrogel (MXene content of 0.4 wt%). MXene hydrogel shows improved mechanical properties compared with pure organohydrogel. As the Gly content increases, the tensile strength and break elongation of MXene organohydrogel considerably increase. The improvement of mechanical properties can be ascribed to the increase in cross-linking density due to the addition of MXene and the displacement of Gly [43, 45, 46]. Figure 2b–d indicates that MXene organohydrogel with considerable hydrogen bonds has excellent deformability and adhesiveness. It can adapt to various shapes, and can be repeatedly torn off and pasted on objects of different shapes. During repeated use, the MXene organohydrogel remains intact without cracks. Additionally, it can lift a bottle with a total weight of 30 g, further revealing its strong adhesiveness. Compared to other MXene-based EMI shielding materials such as MXene aerogels and films, MXene organohydrogel has obvious advantages in convenience and reusability.

In addition, MXene organohydrogel has an attractive self-healing function, which is beneficial to extend its service life in practical applications. Here, the fast and reproducible self-healing ability of the MXene organohydrogel is presented in Fig. 2e, f. Once the two parts of the cut MXene organohydrogel were made into contact, they can be repaired rapidly (~ 3.2 s) under environmental condition (25 °C, humidity 45%). The MXene organohydrogel can be connected into a circuit to power a LED, demonstrating its moderate conductivity. To further verify its self-healing ability, we cut the MXene organohydrogel in half and the LED bulb was turned off. Once the separated organohydrogel was in contact, the LED bulb immediately glowed again. In addition, Fig. S7 shows the change of resistance of MXene organohydrogel during several self-healing processes. When MXene organohydrogel was cutoff, the resistance increased sharply from a steady state to an open circuit state. Subsequently, the resistance rapidly returned to the original value within 3.2 s once the separated pieces were in contact. The self-healing efficiency is defined as the ratio of the break elongation of self-healed MXene organohydrogel to the break elongation of the original MXene organohydrogel. The stress–strain curves reveal that the self-healing efficiency of MXene organohydrogel is above 90% (Fig. S8). The self-healing ability is due to the dynamic cross-linking between the hydroxyl group of PVA and tetrahydroxyl borate ions, and the supramolecular interaction among Gly, PVA and MXene [29].

**Fig. 3:**
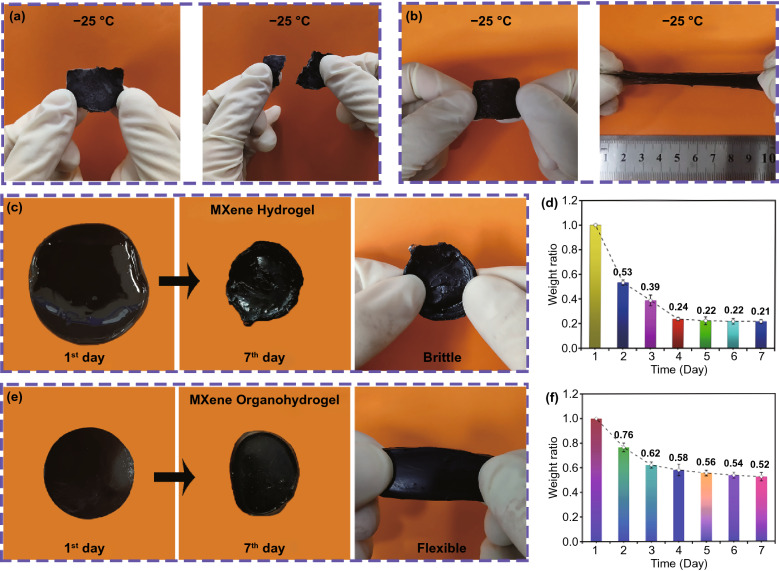
Low-temperature tolerance of **a** MXene hydrogel (25 × 10 × 3 mm^3^) and **b** MXene organohydrogel (original size: 25 × 10 × 3 mm^3^). **c** Photos showing MXene hydrogel on the 1 and 7 days (original size: diameter of ~ 30 mm, thickness of ~ 3 mm; final size: diameter of ~ 20 mm, thickness of ~ 2.7 mm). **d** Change of weight ratio of MXene hydrogel during 7 days of storage. **e** Photos showing MXene organohydrogel on the 1 and 7 days (original size: diameter of ~ 25 mm, thickness of ~ 4 mm; final size: diameter of ~ 20 mm, thickness of ~ 4 mm). **f** Change of weight ratio of MXene organohydrogel during 7 days of storage

In practical applications, the big challenge of MXene hydrogel is to possess long-term environmental stability such as low-temperature tolerance and anti-drying performance. Fortunately, after solvent displacement, abundant hydrogen bonds are formed between water and Gly in MXene organohydrogel, which disrupt the formation of ice crystal below 0 °C and weaken the volatilization of water. Figure 3a, b shows the anti-freezing performances of the MXene hydrogel and organohydrogel. After being stored at − 25 °C for 3 h, the MXene hydrogel was frozen and became brittle, while the MXene organohydrogel still maintained flexibility. Figure 3c–f compares the changes in weight of MXene organohydrogel and hydrogel with storage time under environmental conditions (25 °C, humidity 45%). After storage for 7 days, the volume of the MXene hydrogel obviously shrank, and the weight retention was only ~ 21.7%; while the volume shrinkage of MXene organohydrogel was smaller, and the weight retention was above 50%. SEM images in Fig. S9 show that some pores appear in the MXene hydrogel stored for 7 days due to the significant evaporation of water, while no pores could be observed in the MXene organohydrogel stored for 7 days. The simple one-pot solvent displacement method is feasible for improving the environmental stability of MXene hydrogels. The excellent moisture retention of MXene organohydrogel can be attributed to the lowering of saturated vapor pressure of the mixed solvent and the prevention of water evaporation due to the formation of abundant hydrogen bonds between water and Gly.

### Electrical Conductivity and EMI Shielding Performance

**Fig. 4:**
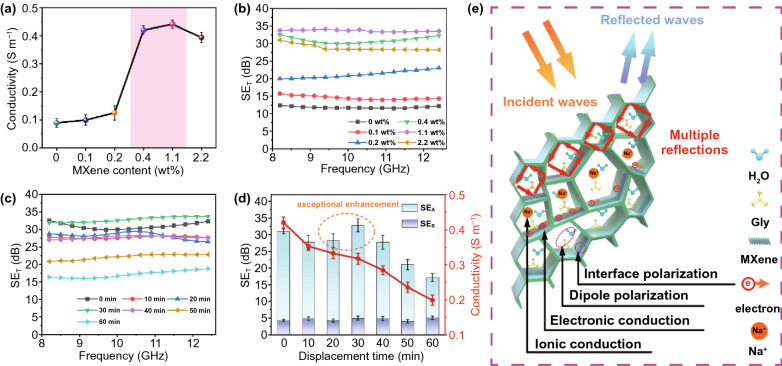
**a** Variation of conductivity of MXene hydrogel with MXene content. **b** EMI SE_T_ curves of MXene hydrogels containing different MXene contents. **c** EMI SE_T_ curves of MXene organohydrogels prepared at different water-Gly (1:1) displacement time. **d** Variations of conductivity and average SE_T_ of MXene organohydrogel with water-Gly displacement time. **e** Scheme showing the EMI shielding mechanism of MXene organohydrogel

Electrical conductivity is generally considered to be the most important factor affecting the EMI shielding performance of materials [13]. The electrical conductivity of MXene hydrogel derives from the migration of free ions (such as sodium ions) via free water inside the polymeric network as well as the transport and hopping of electrons in the polymer networks containing MXene nanosheets. We tested the variation of conductivity of MXene hydrogels with MXene content using four-probe method (Fig. 4a). As MXene content increases from 0.1 to 2.2 wt%, the conductivity of the MXene hydrogel first increases sharply from 0.099 to 0.442 S m^−1^, and then slightly decreases to 0.394 S m^−1^. On the one hand, the increase of MXene content increases the electron transport and hopping paths, thus enhancing electron conduction; on the other hand, the size of ion channels decreases with the increase of MXene content, leading to the reduction of ion conduction. Therefore, the variation of conductivity with MXene content depends on the competition between increased electron conduction and decreased ion conduction.

Highly conductive organohydrogels with high mechanical properties and excellent EMI shielding performance are desirable for deformable and wearable electronic devices. The EMI shielding performance of MXene hydrogels with thickness of 1.0 mm were first evaluated by measuring the scattering parameters (*S*_11_, *S*_12_, *S*_21_, *S*_22_) using a vector network analyzer in the frequency range of 8.2–12.4 GHz. Figure 4b shows the EMI shielding effectiveness (SE) of the MXene hydrogels with different MXene contents. As MXene content increases from 0.1 to 1.1 wt%, the total SE (SE_T_) of the hydrogel first increases from 14.5 to 33.6 dB, and then decreases to 28.7 dB. The highest SE_T_ value is achieved in the MXene hydrogel with the highest conductivity (i.e., containing 1.1 wt% MXene). The change rule of SE_T_ with MXene content is consistent with that of conductivity. If we compare the hydrogels with MXene contents of 0.4 and 1.1 wt%, it is worth noting that when the MXene content is reduced to one third (i.e., 0.4 wt% mg), the SE_T_ only drops by 8%. Therefore, we used the MXene hydrogel with MXene content of 0.4 wt% as a representative in the following study.

MXene organohydrogel with excellent anti-drying and anti-freezing properties is more attractive in practical applications. However, the displacement of water by Gly will reduce the conductivity of the MXene organohydrogel due to the decreased ion transport [23]. As shown in Fig. 4d, the conductivity of MXene organohydrogel decreases from 0.42 to 0.20 S m^−1^ with increasing the displacement time in water-Gly mixture (water:Gly = 1:1). Therefore, the amount of Gly should be carefully controlled to ensure the excellent EMI shielding performance of the MXene organohydrogel. Figure 4c, d shows the SE_T_ of the MXene organohydrogels prepared with different displacement time. Compared with MXene hydrogel with a SE_T_ of 30.8 dB, the MXene organohydrogel with a displacement time of 10 min has a lower SE_T_ value of 27.5 dB. Remarkably, when the displacement time increases to 30 min, the MXene organohydrogel exhibits an exceptionally enhanced EMI shielding performance of 32.8 dB, even higher than that of MXene hydrogel. A further increase of displacement time causes a gradual decrease of SE_T_. To further confirm this abnormal enhancement of EMI shielding ability, we conducted two comparative experiments and immersed MXene hydrogels in water-Gly mixtures with different volume ratios of water and Gly (4:1 and 0:1). Similarly, we also observed the abnormally enhanced shielding performances of MXene organohydrogels in these two experiments (Fig. S10), which once again confirm the non-monotonic increase of EMI SE with conductivity and polarization loss.

The SE_T_ includes the contributions from reflection effectiveness (SE_R_), absorption effectiveness (SE_A_) and multi-reflection effectiveness (SE_M_) [47–49]. Reflection attenuation relies on the impedance mismatch at the interface between air and the shielding materials, and absorption attenuation is associated with the electromagnetic energy conversion through induced current and polarization relaxation [50–52]. As shown in Fig. 4d, SE_A_ makes major contribution to SE_T_. The absorption (*A*) and reflection (*R*) coefficients were calculated for a deep insight into the EM response. As illustrated in Fig. S11, the large average R coefficient indicates the high conductivity, which produces strong reflection to suppress most EM waves from entering the material. Generally, the dissipation pathway within the absorber is described as dielectric loss, through which the EM energy is converted into heat. Dielectric loss is composed of conduction loss and polarization loss [53–55]. In MXene gel, the ion transport through solvent channels and the electron transport along polymer network can produce conduction loss to attenuate EM energy [25, 56–58]. Moreover, water molecules with intrinsic polar property can generate polarization loss through the rotation of dipoles under alternating EM field [25]. If water molecules were displaced by Gly molecules, both conduction loss and polarization loss were weakened because the ion diffusion in water is faster than that in Gly, and the polarization loss of Gly molecules is much lower than that of water molecules in gigahertz frequency due to the differences in molecular weights and polarities [28]. Therefore, a substantial displacement of water by Gly is expected to weaken the EMI shielding ability of MXene organohydrogel. However, when an appropriate amount of Gly displaced water, an abnormal increase in the SE_T_ value was observed, which should be understood in terms of the reflection and scattering of EM waves by the heterogeneous interfaces. As shown in the SEM images in Fig. 1d-f, with the addition of Gly, the pores of the gel become smaller and more compact. The increase in pore density will increase the pore-polymer/MXene interfaces, causing more incident EM waves being scattered/reflected [59–61]. Consequently, more EM waves can be trapped in the porous structure and attenuated to achieve the improvement of the EMI shielding performance. The change of SE_T_ with displacement time depends on the competition of decreased ion conductivity, reduced polarization loss and increased scattering interface. The above experiments highlight the importance of EM wave reflection and scattering in improving EMI shielding performance, which was underestimated previously.

### Environmental Stability of EMI Shielding Performance

**Fig. 5:**
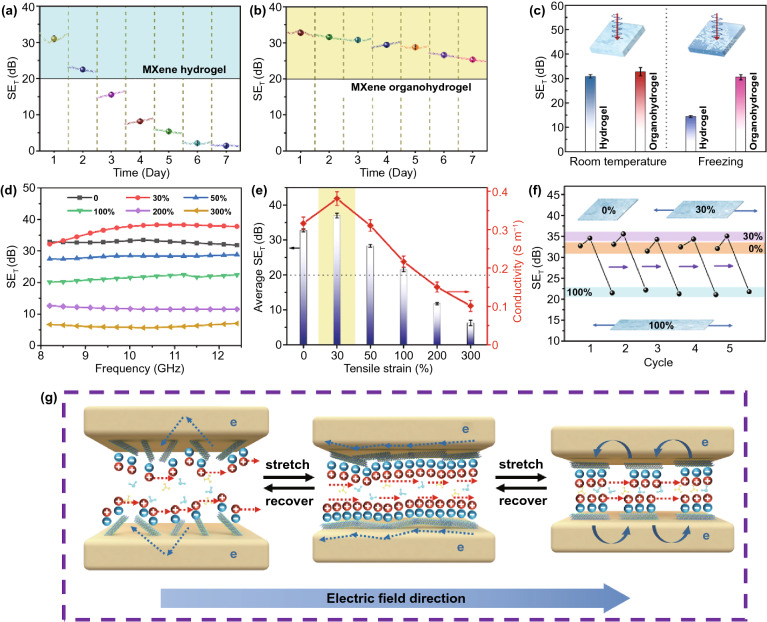
Changes in SE_T_ of **a** MXene hydrogel and **b** MXene organohydrogel with storage time under environmental conditions (25 °C, humidity 45%). **c** Average SE_T_ values of MXene hydrogel and organohydrogel before and after freezing. **d** SE_T_ curves of MXene organohydrogel with different tensile strains. **e** Variations of average SE_T_ value and conductivity of MXene organohydrogel with tensile strain. **f** Average SE_T_ values of MXene organohydrogel with 0%, 30% and 100% strains during five stretching cycles. **g** Scheme illustration of the conducting mechanism of MXene organohydrogel during stretching. The blue negative spheres represent surface charges on MXene sheets, and the red positive spheres represent cations

MXene hydrogel faces the inevitable problems of water evaporation and sub-zero freezing, which will reduce the EMI shielding performances. MXene organohydrogel with excellent anti-drying and anti-freezing properties provides an effective strategy for improving environmental stability. We tested the changes in conductivity and EMI shielding performance of MXene hydrogel and organohydrogel during storage for 7 days (25 °C, humidity 45%). The thickness of MXene hydrogel decreased from 1.0 to 0.9 mm, while the thickness of MXene organohydrogel remained 1 mm. As shown in Fig. S12, the conductivity of MXene hydrogel rapidly decreased from 0.42 to 0.01 S m^−1^, while the conductivity of the MXene organohydrogel only decreased from 0.32 to 0.24 S m^−1^. At the same time, due to higher water evaporation, the polarization loss of MXene hydrogel shows a larger decline than that of MXene organohydrogel. As a result, the SE_T_ value of MXene hydrogel significantly decreased during storage (Fig. 5a), and the SE_T_ retention after 7 days was as low as 5.0% (from 30.8 to 1.54 dB). In contrast, the SE_T_ value of MXene organohydrogel started from 32.8 dB and gradually decreased to 25.3 dB after 7 days, which indicates the high SE_T_ retention of 77.1%, almost 15.5 times of MXene hydrogel. This indicates that the ionic conduction loss and polarization loss of solvent molecules make much more contribution to the attenuation of EM energy than the electron conduction loss.

If the MXene hydrogel was frozen at − 25 °C for 3 h, its SE_T_ decreased from 30.8 to 14.4 dB (Figs. 5c and S13). The sharp drop of more than 50% can be attributed to two reasons. On the one hand, freezing in coldness reduces the amount of free water molecules and thus slows down the ion migration; on the other hand, polar molecules in solid ice are less sensitive to EM waves compared to those in liquid water [25]. Therefore, as the conductivity and polarization relaxation decrease, the reflection and absorption of EM waves by the frozen hydrogel will weaken [62]. Even though suffering from freezing at − 25 ℃ for 3 h, the MXene organohydrogel did not show an obvious decay in shielding performance, and the average SE_T_ value of MXene organohydrogel after freezing still maintained a high value of 30.5 dB. The above experiments show that MXene organohydrogel has excellent environmental stability in terms of drying resistance and low-temperature tolerance, which benefits expanding its applications in harsh environments such as cold northern regions, dry tropical regions and so on.

### Strain Sensitivity of EMI Shielding Performance

Many previous literatures have reported that the conductivity of organohydrogels usually decreases during stretching [23, 30]. This feature is unfavorable for the application of organohydrogel in deformable and wearable electronics, because the decrease in conductivity during stretching will lead to a decrease in shielding performance, which may cause EMI shielding failures when electronics are stretched or bent. Therefore, we checked the variation of average SE_T_ of MXene organohydrogel with tensile strain (Fig. 5d, e). Currently, the SE_T_ of shielding materials for commercial applications is required to be higher than 20 dB. Obviously, the SE_T_ value of MXene organohydrogel is higher than 20 dB in the range of 0–100% tensile deformation, which covers the strain range of most wearable electronics. During the stretching, the thickness of the organohydrogel is reduced from 1.0, 0.98, 0.96, 0.92, 0.83, 0.70 to 0.58 mm.

Interestingly, the SE_T_ value of MXene organohydrogel shows a non-monotonic change within tensile strain of 100%. At a small tensile strain of 30%, the SE_T_ of the organohydrogel increased from 32.8 to 37.0 dB, while the SE_T_ gradually decreased at larger tensile strain. This phenomenon has not been reported in other hydrogel shielding materials [44, 63]. The change trend of SE_T_ is consistent with that of conductivity. To understand the variations of conductivity (SE_T_) with stretching, we observed the microstructure of MXene organohydrogel under different strains (0%, 30%, and 100%). Figure S14 shows the variation of pores of freeze-dried MXene organohydrogel with stretching. As the tensile strain increases, the pores are stretched and aligned in the direction of strain. Figure S15 shows the variation of the distribution of MXene nanosheets in MXene organohydrogel with stretching. The MXene nanosheets randomly distribute in the unstretched hydrogel. When subjected to a small strain (X direction), the MXene nanosheets slid and tend to rearrange in parallel. As the size of the gel decreases in the Y and Z directions, a part of MXene nanosheets can be connected (marked by circles). Under large tensile strain, the MXene nanosheets separate from each other. Therefore, the abnormal enhancement of SE_T_ of MXene organohydrogel under small strain can be ascribed to the following reasons, and the mechanism is illustrated in Fig. 5h. Firstly, the parallel alignment and connection of MXene nanosheets under small strain facilitate the increase of the electron conduction paths. Secondly, since more MXene planes are parallel to the direction of electric field vector under small strain, a large number of mobile charge carriers in MXene planes could collectively induce a stronger internal electric field to partially offset the external EM field and convert more EM energy into heat. Thirdly, the rearrangement of the MXene nanosheets under small strain can change the ionic affinity of the organohydrogel pores, which facilitates tuning the ionic transport. When MXene hydrogel is stretched under small strain, the stretched pores provide narrower spacing, wherein the surface charge-induced cation transport is developed, thus increasing ionic conductance in the confined pores [64].

In addition, we tested the stability of SE_T_ during cycling stretching. As presented in Fig. 5f, the MXene organohydrogel with 0%, 30%, and 100% strains did not show a significant decay of SE_T_ value during five stretching cycles, which demonstrates its good performance stability. With an unprecedented combination of high EMI shielding ability, unusual strain-enhanced shielding performance, outstanding deformability, self-healing ability, environmental stability, skin-compatible mechanical properties, and facile preparation, our MXene organohydrogel has potential applications in providing EMI protection for deformable and wearable electronic devices.

## Conclusion

In summary, we developed MXene organohydrogel containing water and Gly binary solvents, which has excellent stretchability, shape adaptability and environmental stability, rapid self-healing ability and efficient EMI shielding ability. Surprisingly, the MXene organohydrogel exhibited two unusual EMI shielding enhancement phenomena. On the one hand, by adjusting the Gly-water ratio in gel, the shielding effectiveness of MXene organohydrogel was abnormally enhanced relative to MXene hydrogel due to the increase in the density of polymer network. On the other hand, when the MXene organohydrogel was slightly stretched, the parallel orientation and connection of MXene nanosheets lead to the abnormal enhancement of shielding effectiveness. This work provides an effective strategy to overcome the bottleneck of hydrogels used as EMI shielding materials, that is, obtaining excellent environmental stability (drying resistance and freezing tolerance) while maintaining highly efficient EMI shielding performance. In addition, the important role of increasing cross-linking density of gel to improve EMI shielding performance was revealed. The prepared MXene organohydrogel has the potential to provide EM radiation protection for deformable and wearable electronic devices. At the same time, this work also expands the applications of MXene organohydrogels.

## Supplementary Information

Below is the link to the electronic supplementary material.

Supplementary file1 (DOCX 2984 kb)
