# Supplementary material for: Environmentally Tough and Stretchable MXene Organohydrogel with Exceptionally Enhanced Electromagnetic Interference Shielding Performances
Source: Nanomicro Lett. 2022 Mar 21;14:77. doi: 10.1007/s40820-022-00819-3 (PMC8938570; doi:10.1007/s40820-022-00819-3)
Supplement: Supplementary file 1 — Supplementary file1 (DOCX 2984 kb) [file 40820_2022_819_MOESM1_ESM.docx]

Supplementary Information

Environmentally Tough and Stretchable MXene Organohydrogel with Exceptionally Enhanced Electromagnetic Interference Shielding Performances

Yuanhang Yu^1#^, Peng Yi^1#^, Wenbin Xu^2^, Xin Sun^1,3^, Gao Deng^1^, Xiaofang Liu^1,^*, Jianglan Shui^1^, Ronghai Yu^1^

^1^School of Materials Science and Engineering, Beihang University, Beijing, 100191, P. R. China
*Corresponding author. E-mail address: liuxf05@buaa.edu.cn (X. Liu).

^2^Science and Technology on Optical Radiation Laboratory and ^3^Science and Technology on Electromagnetic Scattering Laboratory, Beijing Institute of Environmental Features, Beijing 100854, P. R. China


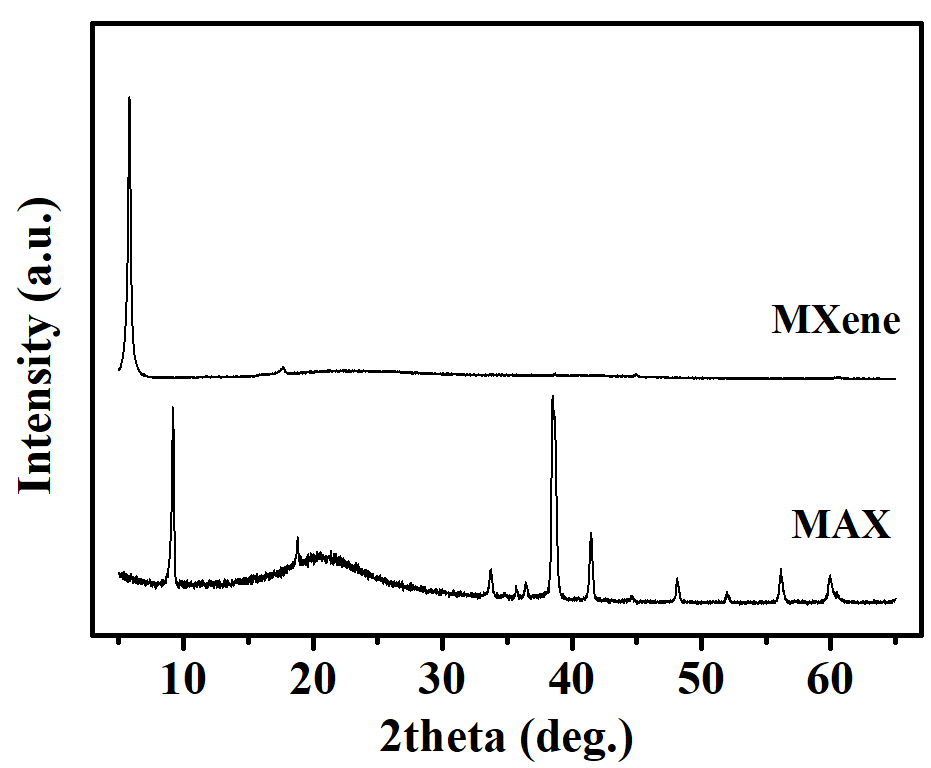


Fig. S1 XRD patterns of MAX powder and MXene nanosheets.


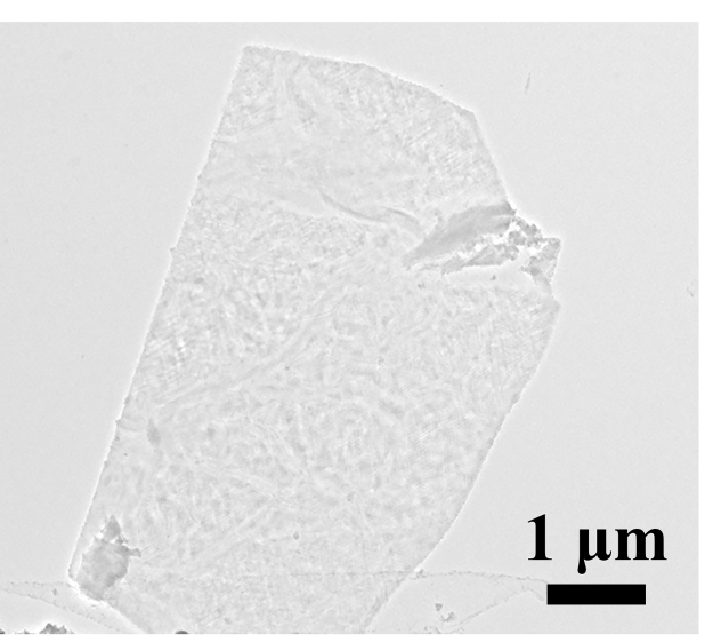


Fig. S2 TEM image of MXene nanosheet.


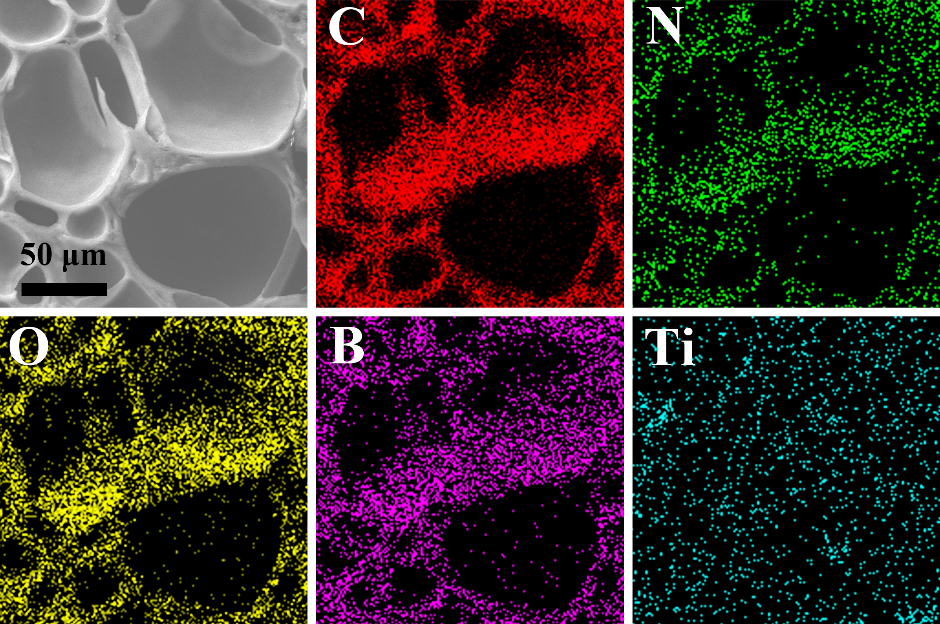


Fig. S3 SEM and elemental mapping images of MXene hydrogel (MXene content of 0.4 wt%).


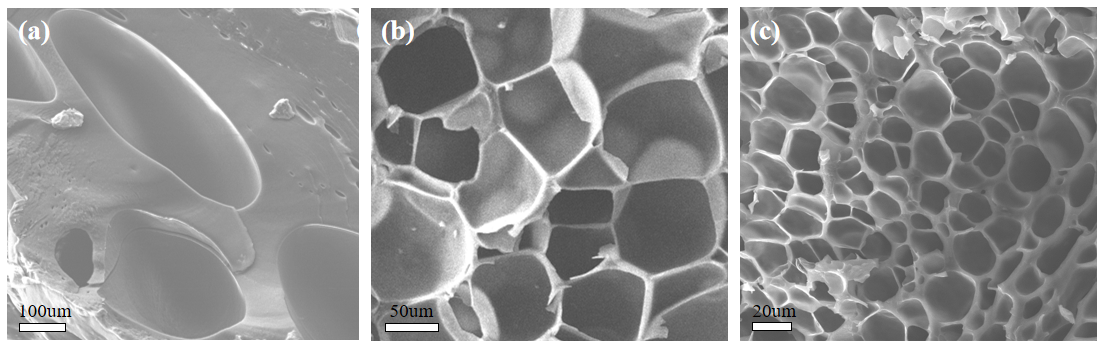


Fig. S4 SEM images of (a) pure hydrogel, (b) MXene hydrogel with MXene content of 0.4 wt%, and (c) MXene hydrogel with MXene content of 2.2 wt%.


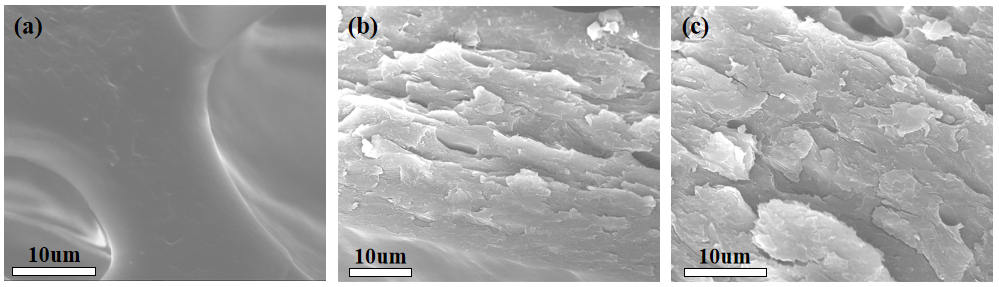


Fig. S5 High magnification SEM images of (a) pure hydrogel, (b) MXene hydrogel with MXene content of 0.4 wt% and (c) MXene hydrogel with MXene content of 2.2 wt%.


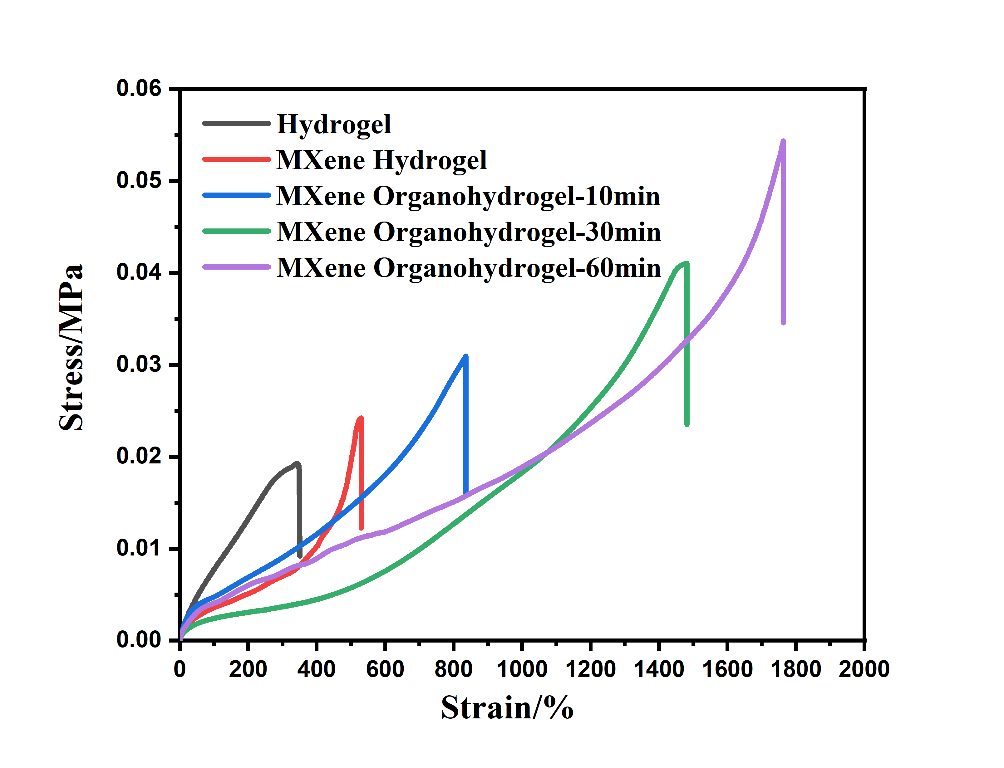


Fig. S6 Stress-strain curves of pure hydrogel, MXene hydrogel and MXene organohydrogels.


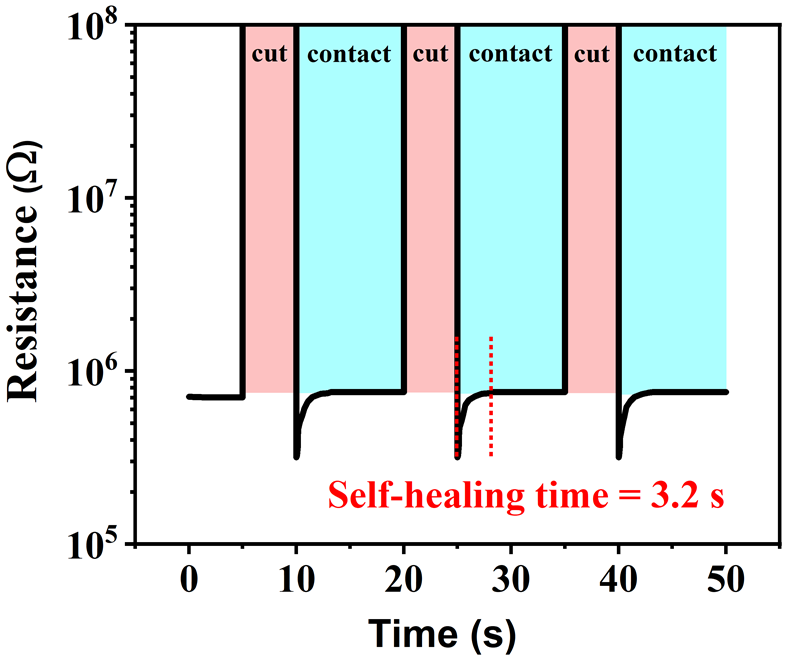


Fig. S7 Change of resistance of MXene organohydrogel during several self-healing processes.


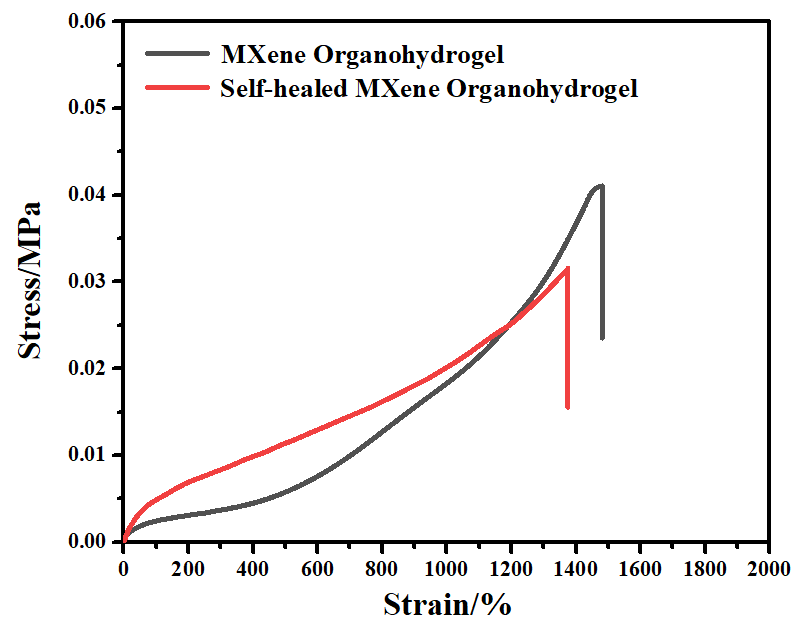


Fig. S8 Stress-strain curves of original and self-healed MXene organohydrogels.


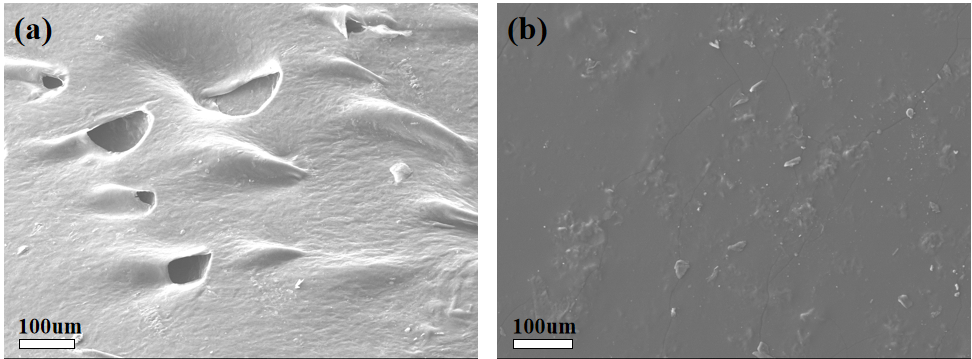


Fig. S9 SEM images of (a) MXene hydrogel stored for 7 days and (b) MXene organohydrogel stored for 7 days.


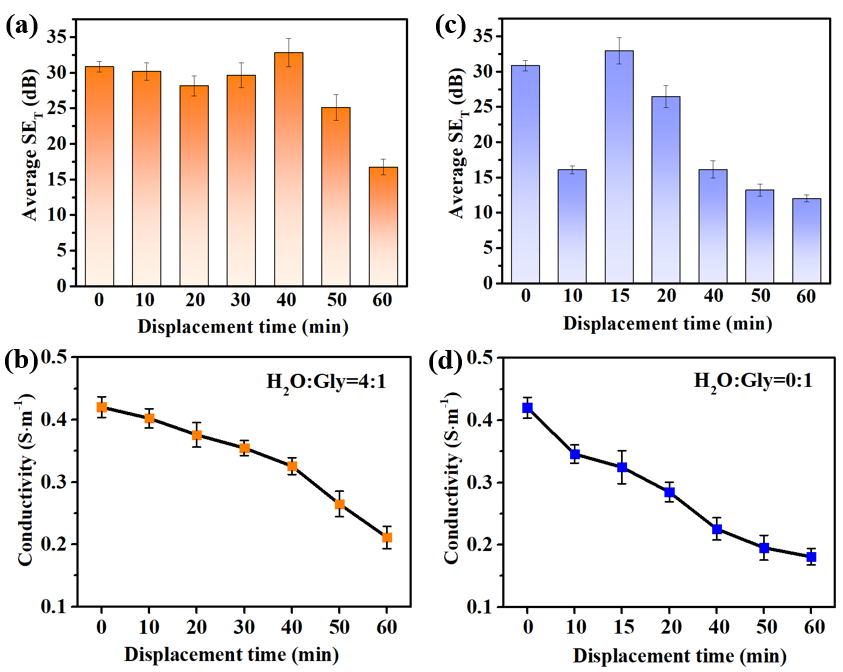


Fig. S10 Variations of (a) average SE_T_ and (b) conductivity of MXene organohydrogel (water:Gly=4:1) with displacement time. Variations of (c) average SE_T_ and (d) conductivity of MXene organohydrogel (water:Gly=0:1) with displacement time.


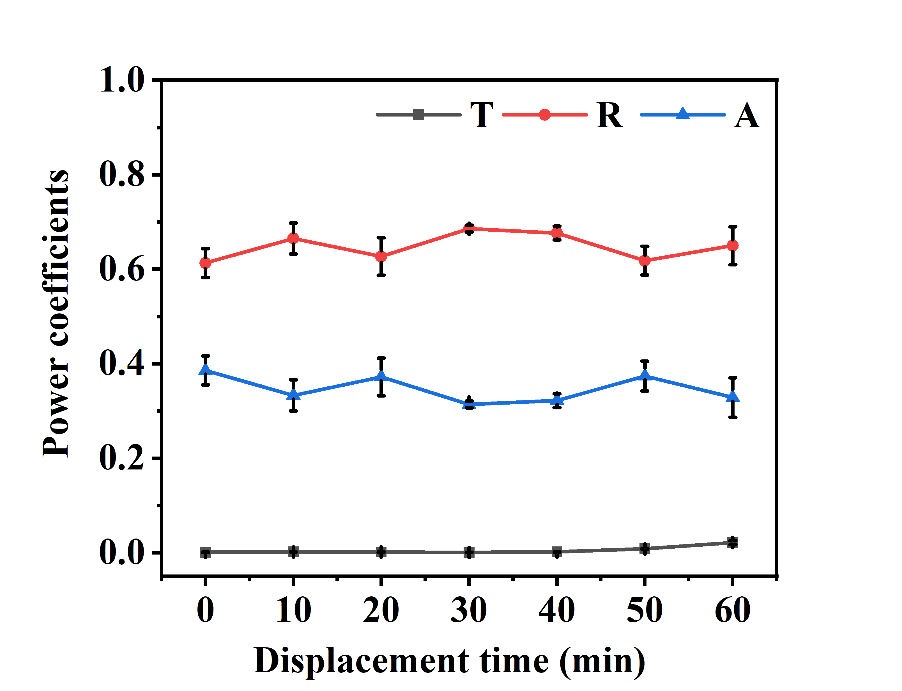


Fig. S11 The absorption (A), reflection (R) and transmission (T) coefficients of MXene organohydrogels with different water-Gly (1:1) displacement time.


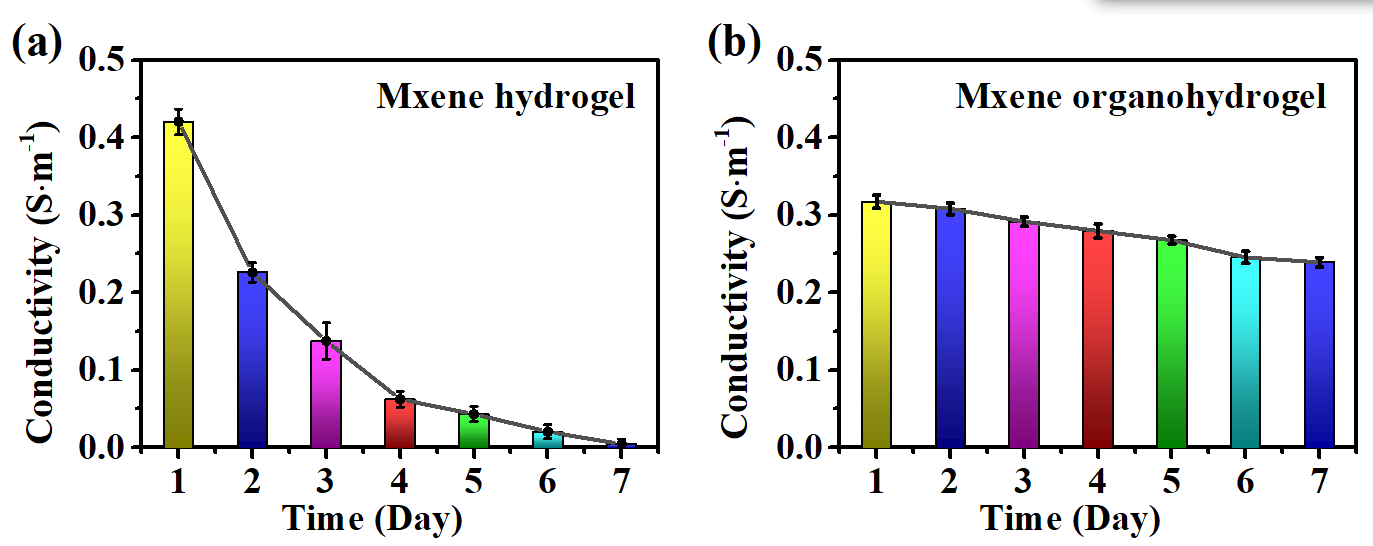


Fig. S12 Variations of conductivity during storage: (a) MXene hydrogel, (b) MXene organohydrogel.


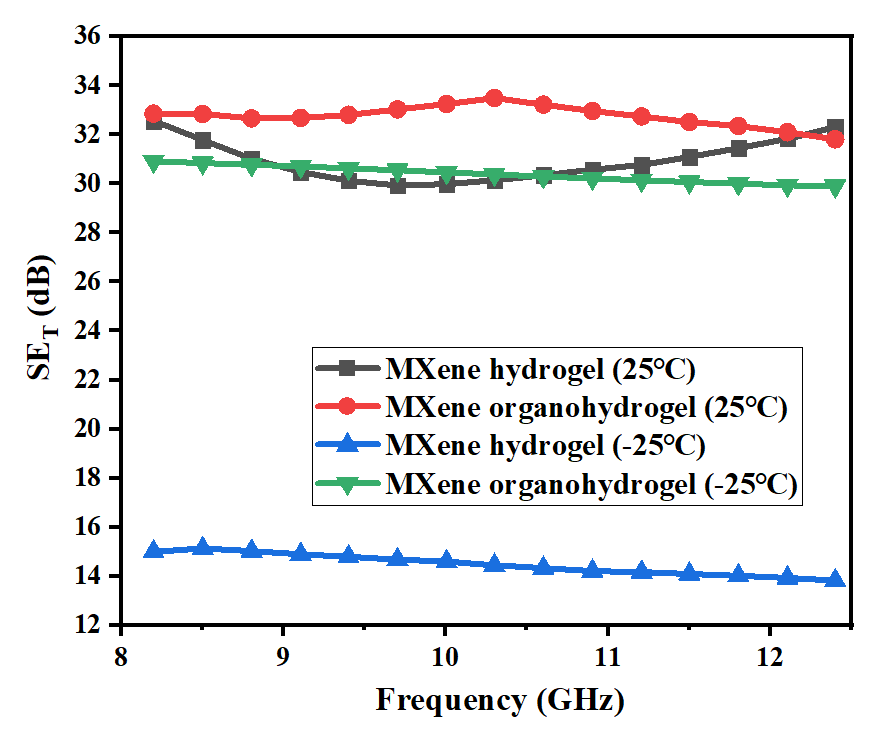


Fig. S13 SE_T_ curves of MXene hydrogel and MXene organohydrogel before and after freezing.


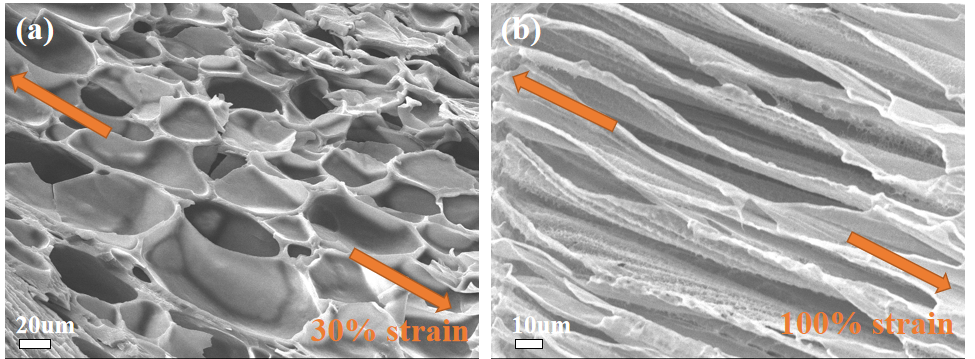


Fig. S14 SEM images of freeze-dried MXene organohydrogels (MXene content of 0.4 wt%, Gly displacement time of 30 min) under (a) 30% and (b) 100% strains.


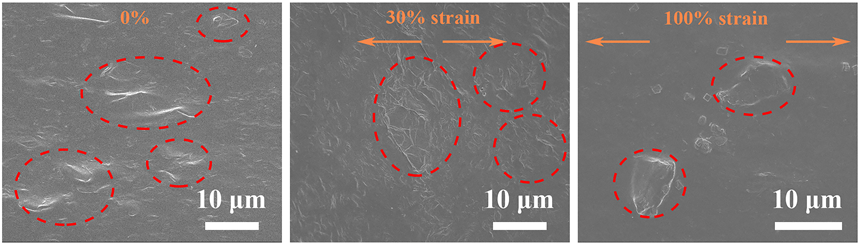


Fig. S15 SEM images of MXene organohydrogel under 0%, 30% and 100% strains.
